# Breast milk concentrations of acetaminophen and diclofenac - unexpectedly high mammary transfer of the general-purpose drug acetaminophen

**DOI:** 10.1186/s12884-024-06287-4

**Published:** 2024-01-29

**Authors:** Ryo Tamaki, Kiwamu Noshiro, Ayako Furugen, Ayako Nishimura, Hiroshi Asano, Hidemichi Watari, Masaki Kobayashi, Takeshi Umazume

**Affiliations:** 1 Department of Obstetrics, Hokkaido University Hospital, Kita-ku N15 W7, Sapporo, 060-8638 Japan; 2 Laboratory of Clinical Pharmaceutics & Therapeutics, Division of Pharmasciences, Faculty of Pharmaceutical Sciences, Hokkaido University, Sapporo, Japan; 3 Department of Pharmacy, Hokkaido University Hospital, Sapporo, Japan; 4 Education Research Center for Clinical Pharmacy, Faculty of Pharmaceutical Sciences, Hokkaido University, Sapporo, Japan

**Keywords:** Acetaminophen, Breastfeeding, Diclofenac sodium, Liquid chromatography-electrospray ionization tandem mass spectrometry, Mammary transfer, Milk-to-plasma ratio

## Abstract

**Background:**

Breastfeeding is considered to be the most effective way of ensuring the health and survival of newborns. However, mammary transfer of drugs administered to mothers to breastfeeding infants remains a pressing concern. Acetaminophen and diclofenac sodium are widely prescribed analgesics for postpartum pain relief, but there have been few recent reports on the mammary transfer of these drugs, despite advances in analytic techniques.

**Methods:**

We conducted a study on 20 postpartum mothers from August 2019–March 2020. Blood and milk samples from participants were analyzed using liquid chromatography-electrospray ionization tandem mass spectrometry within 24 hours after oral administration of acetaminophen and diclofenac sodium. The area under the concentration-time curve (AUC) was calculated from the concentration curve obtained by a naive pooled-data approach.

**Results:**

For acetaminophen, AUC was 36,053 ng/mL.h and 37,768 ng/mL.h in plasma and breast milk, respectively, with a milk-to-plasma drug concentration ratio of 1.048. For diclofenac, the AUC was 0.227 ng/mL.h and 0.021 ng/mL.h, in plasma and breast milk, respectively, with a milk-to-plasma drug concentration ratio of 0.093.

**Conclusions:**

While diclofenac sodium showed low mammary transfer, acetaminophen showed a relatively high milk-to-plasma drug concentration ratio. Given recent studies suggesting potential connections between acetaminophen use during pregnancy and risks to developmental prognosis in children, we believe that adequate information regarding the fact that acetaminophen is easily transferred to breast milk should be provided to mothers.

## Background

Breastfeeding is a matter of nutritional concern for infants and has many advantages for both mother and infant. For the infant, it has a protective effect against infections such as upper respiratory tract infections and gastrointestinal diseases after infancy [1], and for the mother, the delay in the resumption of menstruation due to breastfeeding reduces the risk of breast and ovarian cancer [2]. On the other hand, there are also drawbacks to breastfeeding. Breast milk is less effective than artificial milk in stimulating intestinal growth [3], and the widespread obsession with breastfeeding supremacy may induce postpartum depression [4]. Furthermore, although breast milk has many positive effects on mother and child, the mammary transfer of drugs administered postpartum is often a concern.

The amount of a drug that is transferred into breast milk is difficult to accurately assess because of the interactive effects of drug liposolubility, molecular size, concentration in the maternal circulation, protein binding capacity, infant and maternal bioavailability, and drug half-life, but its safety is evaluated using indicators such as relative infant dose (RID) and milk-to-plasma drug concentration ratio (M/P ratio) [5]. However, all patients have individual differences, for example, abnormalities in renal or hepatic function on the part of the mother are one of the causes of high drug concentrations in breast milk [6], and there is a wide range of variations in the mammary transfer of drugs. For example, in cases of mastitis, the permeability between the lumen in the mammary gland and the blood is increased due to the disruption of the blood-milk barrier, the “alveolar epithelial tight junctions” [7]. Within the first 72 hours postpartum, the alveolar epithelial tight junctions loosen and the amount of drugs, immunoglobulins, maternal cells (lymphocytes, leukocytes, macrophages), and other maternal proteins that pass through the blood-milk barrier increase [5]. This initial secretion is known as colostrum, and by the time 1 week postpartum has passed, prolactin causes the alveolar epithelial cells to enlarge and the blood-milk barrier to tighten, reducing the mother’s secretion of drugs, proteins, and other substances. Thus, in addition to individual differences, the transfer of drugs between plasma and breast milk varies with the condition of the mammary gland and the timing of milk secretion.

In the postpartum period, pain in the perineum or Cesarean section wound, headaches, infections, postpartum depression, or recurrence of pre-existing illnesses is observed, often requiring analgesics, antibiotics, antidepressants, and other medications. When a nursing mother needs medication, we often consult LactMed database [8] to determine if the drug can be administered during lactation. One of the most common postpartum complaints is pain, and common analgesics such as acetaminophen, diclofenac sodium, and ibuprofen are considered safe drugs [9]. There are several reports on the effects of maternal analgesics on neonates [10–12]. Asthma and rashes have been reported when infants drink large amounts of acetaminophen in breast milk [10, 11]. It has also been reported that neonates of women who took diclofenac during pregnancy developed renal failure [12]. Therefore, data on the amount of analgesics transferred into breast milk are important, but recent studies are scarce. As far as we could find, there have been only four reports on acetaminophen concentrations in breast milk since 1980 [13–16]. There were also only three reports since 1970 on mammary transfer of diclofenac sodium, which is often administered similarly to acetaminophen [17–19]. Most of these were case reports.

The last report on mammary transfer was in 1987 for acetaminophen and in 1988 for diclofenac sodium, and despite advances in measuring equipment, there have been no recent reports. For diclofenac, many details remain unknown. We felt that in order to ensure breastfeeding safety of acetaminophen and diclofenac sodium, which are used by many nursing mothers, it would be necessary to reexamine their mammary transfer. In this study, we used a quantitative drug concentration assay using liquid chromatography-tandem mass spectrometry, to examine the transfer of acetaminophen and diclofenac sodium into breast milk in 20 women after childbirth.

## Methods

Subjects included those who gave birth at Tenshi Hospital (Sapporo, Japan) between August 2019 and March 2020, used acetaminophen and diclofenac sodium as analgesics postpartum, and breastfed. This study was approved by the Clinical Research Ethics Committee of Hokkaido University Hospital (No. 018–0048) and the Ethics Committee of Tenshi Hospital (No. 98). Written consent was obtained from all patients participating in the study.

Pregnant women with abnormal renal or hepatic function prior to delivery, gestational diabetes mellitus, or complications other than thyroid dysfunction were excluded. The timing of oral administration of acetaminophen and diclofenac sodium was optional, according to patient preference. The blood and breast milk samples for the study were collected at the same time during the postpartum blood draws scheduled in advance in routine practice, from 3 to 6 days postpartum. The time elapsed from the last acetaminophen 500 mg oral tablet, diclofenac sodium 25 mg oral tablet, or diclofenac sodium 50 mg suppository to the blood collection scheduled in routine practice was recorded. The plasma and breast milk samples obtained were stored at − 80 °C until measurement.

### Sample (protein removal procedure)

Before measurement, 10 μL of an Internal Standard (1 μg/mL acetaminophen-d_4_ and diclofenac-d_4_ in methanol) and 800 μL of acetonitrile were added to 200 μL of breast milk or plasma and centrifuged at 13,000×g for 10 minutes at 4 °C. The supernatant (800 μL) was then collected and dried with N_2_ gas at 40 °C. A total of 100 μL of a 10 mM ammonium acetate and methanol 85:15, v/v solution was added to this and the resulting mixture was filtered through a DISMIC-13HP filter to remove particles.

### Quantification and analysis

Measurements were made using liquid chromatography-electrospray ionization tandem mass spectrometry (LC-MS/MS). Chromatographic separation was performed using a Shimadzu Prominance 20 A System (Shimadzu, Kyoto, Japan) and an Inertsustain phenyl-hexyl column (2.0 × 150 mm, 3 μm GL Science Inc., Tokyo, Japan). A binary mobile phase consisted of methanol containing 0.1% acetic acid and 10 mM ammonium acetate solution containing 0.1% acetic acid was flown at a rate of 0.2 mL/min. The methanol composition of the mobile phase was increased from 15 to 90% in a linear gradient over 2 min and maintained at 90% for the first 7.0 min. Methanol composition was then decreased to 15% from 7.0 min to 8.0 min and maintained at 15% until 13 min. The column temperature was maintained at 40 °C. The total run time was 13 min. Positive ion electrospray (ESI)- tandem mass analysis was performed using an API 3200™ LC/MS/MS System with multiple reaction monitoring (MRM) (Applied Biosystems, Foster City, CA). Ion transitions monitored were *m/z* 152.2 → 110.1 for acetaminophen, *m/z* 156.2 → 114.0 for acetaminophen-d_4_, *m/z* 296.1 → 214.0 for diclofenac, and *m/z* 300.0 → 218.2 for diclofenac-d_4_. Parameter settings were as follows: source temperature of 650 °C, spray voltage of 5500 V, curtain gas of 55 psi, ion source gas1 of 50 psi, ion source gas2 of 80 psi, and collision gas of 55 arbitrary units. Data were acquired and analyzed using Analyst software (Applied Biosystems). Lower limit of quantification of acetaminophen and diclofenac were 1 ng/mL and 0.5 ng/mL, respectively. Concentration curves by a naive pooled-data approach were prepared for acetaminophen and diclofenac sodium for 0–24 hours after oral administration using the JMP Pro16© [20, 21].The area under the curve (AUC) for breast milk concentration and plasma concentration was calculated using the WinNonlin© and calculate the milk to plasma drug concentration ratio (M/P ratio).

## Results

A total of 20 puerperant women participated in the study, of which 18 were taking acetaminophen and 15 were taking diclofenac.

### Demographic characteristics

Of the 20 study participants, 10 (50%) were first-time mothers and 17 (85%) had Cesarean sections. Three participants responded to the collection of two blood and milk samples. The age and gestational age of the participants was 35.3 ± 4.2 years and 38.0 ± 1.2 weeks, respectively. The timing of blood collection was 5.6 ± 0.9 days postpartum. Data were obtained for acetaminophen in 20 cases (18 patients) and for diclofenac in 17 cases (15 patients) (Table 1).

**Table 1:** Demographic characteristics of 20 women

Nulliparous women	10 (50%)
Age (years)	35.3 (4.2)
Height (m)	1.59 (0.05)
Body weight (kg)	64.0 (9.5)
Body mass index (kg/m^2^)	25.2 (3.5)
Gestational week at delivery (week)	38. 0 (1.2)
Preterm delivery	4 (20%)
Vaginal delivery	3 (15%)
Cesarean delivery	17 (85%)
Blood loss at delivery (g)*	648 (323)
Timing of tests (Postpartum day)	5.6 (0.9)

Data are presented as the means (standard deviation). *Measured by the amount of suction and the change in the weight of the absorbent mat and gauze

### Pharmacokinetics of acetaminophen

**Fig. 1:**
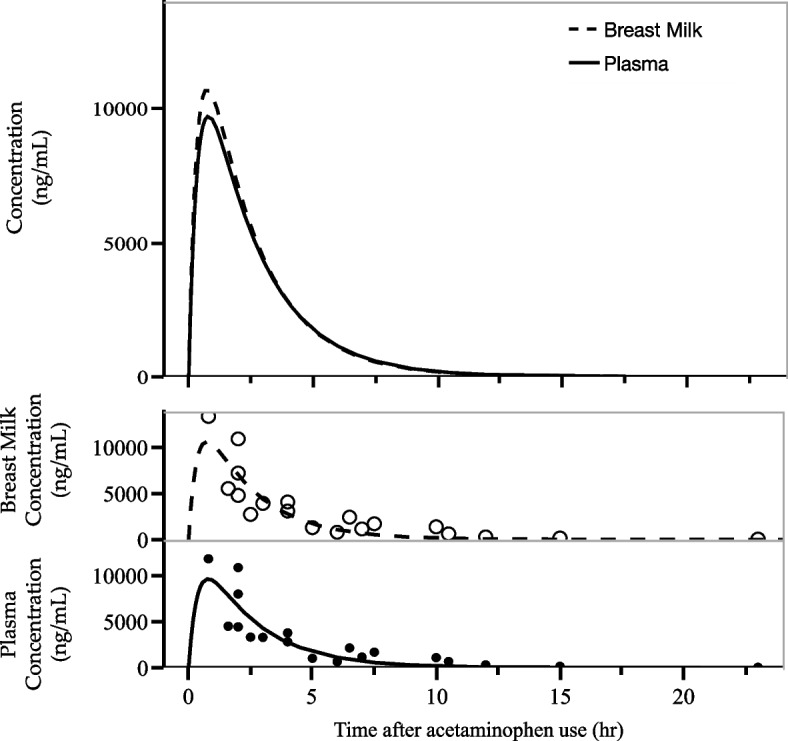
The concentration of acetaminophen in plasma and breast milk. The top graph shows overlaid plasma and breast milk concentrations over time after acetaminophen use, while the bottom graph plots them separately

After oral administration of 500 mg of acetaminophen, the maximum concentration in plasma was 11,920 ng/mL and the AUC was 36,053 ng/mL.h. In breast milk, the maximum concentration was 13,480 ng/mL and AUC was 37,768 ng/mL.h. The M/P ratio was 1.048 (Fig. 1, Table 2).

**Table 2:** Data of 18 women who used acetaminophen

Patient number	Body-Mass Index (kg/m^2^)	acetaminophen dose (mg)	Days after delivery	Time after acetaminophen use (hr)	Plasma concentration (ng/ml)	Breast milk concentration (ng/ml)
1	19.6	500	6	15	165	186
1	19.6	500	6	23	57.5	74.3
2	24.8	500	6	10	1108	1406
3	23.2	500	6	6	677	815
4	31.3	500	6	68	2.31	2.51
5	22.2	500	6	7.5	1710	1740
6	27.7	500	6	3	3330	3970
7	28.0	500	6	5	1040	1320
8	18.9	500	6	2	10,960	11,020
9	23.0	500	6	2	4480	4840
10	29.4	500	4	1.6	4550	5590
11	28.0	500	6	2.5	3350	2770
12	27.0	500	6	12	328	305
13	26.4	500	4	2	8070	7290
13	26.4	500	4	4	3820	4120
14	20.5	500	6	6.5	2170	2460
15	21.1	500	6	0.8	11,920	13,480
16	28.7	500	6	7	1170	1170
17	28.9	500	6	4	2850	3130
18	25.7	500	6	10.5	690	643

### Pharmacokinetics of diclofenac sodium

**Fig. 2:**
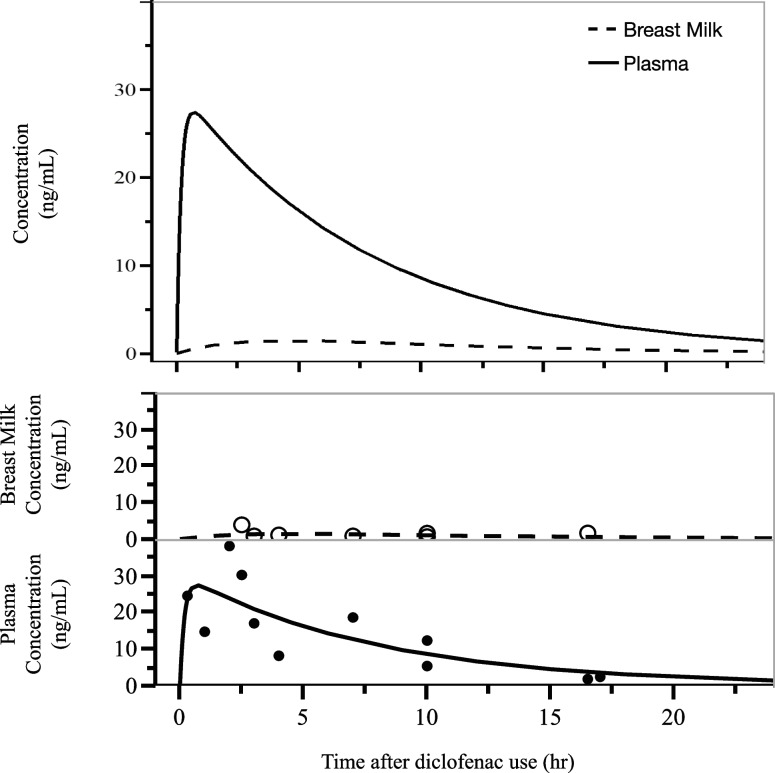
The concentration of diclofenac in plasma and breast milk. The top graph shows overlaid plasma and breast milk concentrations over time after diclofenac sodium use, while the bottom graph plots them separately

In the 10 participants who took oral diclofenac sodium 25 mg and four participants who used a diclofenac sodium 50 mg suppository, the maximum concentration in plasma was 38.1 ng/mL and the AUC was 0.227 ng/mL.h. In breast milk, the maximum concentration was 3.89 ng/mL and the AUC was 0.021 ng/mL.h. The M/P ratio was 0.093 (Fig. 2, Table 3).

**Table 3:** Data of 15 women who used diclofenac

Patient number	Body-Mass Index(kg/m^2^)	Diclofenac dose (mg)	Days after delivery	Time after diclofenac use (hr)	Plasma concentration (ng/mL)	Breast milk concentration (ng/mL)
1	19.6	25	6	3	17	0.84
1	19.6	25	6	2	38.1	N.D.
3	23.2	25	6	17	2.38	N.D.
5	22.2	25	6	23	N.D.	N.D.
6	27.7	25	6	0.3	24.5	N.D.
7	28.0	50	6	16.5	1.78	1.63
8	18.9	25	6	10	5.34	1.58
10	29.4	50	4	10	12.3	0.655
11	28.0	25	6	19	N.D.	N.D.
12	27.0	50	6	7	18.6	0.824
14	20.5	25	6	64	N.D.	N.D.
15	21.1	25	6	64	N.D.	N.D.
16	28.7	50	6	54	N.D.	N.D.
18	25.7	25	6	42	N.D.	N.D.
19	23.4	25	5	2.5	30.2	3.89
20	25.6	25	3	1	14.7	N.D.
20	25.6	25	3	4	8.15	1.1

*N.D*. Not Detected

### Associations of plasma and milk drug concentrations

**Fig. 3:**
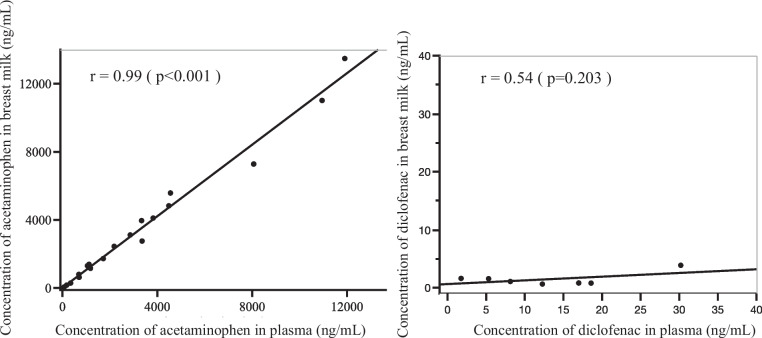
Associations of plasma and milk drug concentrations. Plots show concentrations of maternal plasma and breast milk collected at the same time

We examined the relationship between maternal plasma and breast milk concentrations collected at the same time. The plasma concentrations correlated with breast milk concentrations at r = 0.99 (*p* < 0.001) for acetaminophen, but for diclofenac sodium was not correlated at r = 0.54 (*p* = 0.203) (Fig. 3).

## Discussion

In this study, plasma and breast milk were evaluated using a quantitative drug concentration assay using liquid chromatography-tandem mass spectrometry, and it was found that the M/P ratio of acetaminophen was as high as 1.048 at 5.6 ± 0.9 days postpartum, while the M/P ratio of diclofenac sodium was 0.093, indicating low mammary transfer.

Factors that determine mammary transfer on the drug side include the molecular weight of the drug, protein binding, liposolubility, oral bioavailability to the infant and mother,and drug half-life [5, 6]. In general, the lower the molecular weight of a drug, the more likely it is to be transferred into breast milk. This is because diffusion through the alveolar epithelial cells is much easier. Drugs with molecular weights less than 500 are particularly easy to transfer, while those with molecular weights greater than 800 are less likely to be transferred [22]. Drugs that are highly liposoluble are transferred easily, and many of them are present in maternal plasma bound to albumin. Low plasma protein binding results in a free component of the drug that transfers into milk through the blood-milk barrier. Therefore, it is always desirable to select drugs with high plasma protein binding rates. When a drug passes from the mother to the milk and is ingested by the infant, it is absorbed in the infant’s gastrointestinal tract. Many drugs are absorbed orally at a slow rate, and only a few are absorbed by the infant due to the denaturation of the drug by gastric acid. It is believed that even if absorbed, not many of these drugs are broken down in the liver and reach the infant’s circulating plasma [23]. Furthermore, preference should be given to drugs with short drug half-lives.

Acetaminophen is a drug with a molecular weight of 151.16 and a plasma protein binding rate of 24% [24]. Drugs of molecular weights below 300 are considered small and show increased mammary transfer [25]. In other words, acetaminophen was thought to have accelerated mammary transfer due to its low molecular weight and low plasma protein binding rate. Previous reports indicate that acetaminophen concentrations in plasma and breast milk reach a peak after 1–2 hours of oral administration and then decrease [16]. In our study, the highest acetaminophen concentration in breast milk was 13,480 ng/mL and the highest in plasma was 11,920 ng/mL, so the concentration in breast milk was higher. In the study by Notarianni et al., the mean AUC for breast milk concentration was 21,100 ng/mL.h, while the mean AUC for plasma concentration was 13,080 ng/mL.h, with the breast milk concentration being higher [15]. The antacid cimetidine, a histamine H2 receptor antagonist, is known to be actively transported into milk [26]. Mammary epithelial transport processes have been studied in rats, suggesting multiple active transport systems [27]. Although active transport of acetaminophen in the mammary epithelium has not yet been reported, it is possible that there is an active transport system in the mammary epithelium of acetaminophen, given that several reports, including ours, have reported higher levels of acetaminophen in milk than in plasma (Figs. 1 and 3).

Diclofenac sodium is a drug with a molecular weight of 318.13 (296.1 because it dose not bind to sodium in the body) and a plasma protein binding rate of 99.5% [28]. There have been only a few case reports and no systematic studies on diclofenac transfer to breast milk, all of which reported that diclofenac sodium was barely detectable in breast milk [8, 29]. It was stated that diclofenac sodium was not detected in breast milk even after 50 or 100 mg of diclofenac sodium per day was given for 1 week, and in mice, only 0.2% of the plasma concentration was detected in breast milk [28]. Todd et al. reported that when nursing mothers were given 100 mg of diclofenac per day, no diclofenac was detected in breast milk, but 100 μg/L was detected in breast milk when diclofenac was administered at 150 mg per day [18]. In our study as well, diclofenac sodium was barely detected in breast milk (Figs. 2 and 3). For pregnant women using diclofenac sodium 25 mg orally and 50 mg suppositories, diclofenac sodium was scarcely detected in breast milk for either dosage. Loxoprofen sodium, one of the other non-steroidal anti-inflammatory drugs often used postpartum like diclofenac, has a molecular weight of 304.31, a plasma protein binding rate of 97%, and peaks at 1.5 hours in plasma but was reportedly not detected in breast milk [30].

The amount of acetaminophen transferred to breast milk is 1.3 ~ 4.8% of the mother’s oral dose [10], which is sufficiently low compared with the standard dose of 140 mg/kg for acetaminophen poisoning in children [31]. But there have been reports of skin eruptions in newborns who drank breast milk from women taking acetaminophen [10] and an increase in asthma in infants of nursing women who frequently took acetaminophen [11]. In this way, short-term side effects of unknown mechanism are recognized, even if they are not poisoning, and long-term side effects are not yet well understood. In recent years, fetal exposure to acetaminophen during pregnancy has been associated with a higher probability of attention-deficit/hyperactivity disorder (ADHD) and autism spectrum symptoms [32]. ADHD is one of the most common neurobehavioral disorders, affecting more than 5–10% of the school-age population and is characterized by inattention, hyperactivity, and impulsivity [33]. ADHD is most commonly diagnosed and treated between the ages of 7 and 12 [34]. It is reported to persist into adolescence and beyond in approximately 80% of cases [35]. The rapid increase in neurodevelopmental disorders in children, including ADHD, which has become a problem in recent years [36], could of course be due to societal changes, but on the other hand, we as medical professionals have a responsibility to find its cause in the field of medicine.

Compared to acetaminophen, which transfers through the placenta during pregnancy, the effect of acetaminophen due to breastfeeding is considered low due to bioavailability. However, we do not believe that the effects of acetaminophen in breast milk on the child should be underestimated, just as the careless administration of acetaminophen to the mother during pregnancy, which is suspected to be associated with the risk of ADHD, should be avoided. The data that assure the safety of acetaminophen and diclofenac sodium used postpartum for breastfeeding have not been updated since the 1980s. This study shows that acetaminophen has a very good breast milk transfer. This does not necessarily mean that acetaminophen should be avoided during lactation, but accurate information should be provided to pregnant women.

This study has several limitations. First, we did not obtain a RID, a common measure of breastfeeding safety. We did not calculate RID because of the difficulty of measuring infant feeding volume, which is necessary when calculating RID, and the issue with the reliability of estimating infant feeding volume using infant weight change as an indicator. Second, the data used to determine the M/P ratio from the AUC were obtained in a cross-sectional manner, and it is preferable to use data obtained repeatedly from a single subject. However, we did not choose a highly invasive method and prioritized the quality of life of the subjects. Third, the sample size was small, albeit large compared to the scale of previous studies. Systematic studies of larger populations are needed in the future.

## Conclusion

While diclofenac sodium showed low mammary transfer, acetaminophen showed a relatively high milk-to-plasma drug concentration ratio. Although an association between infant acetaminophen intake via breast milk and long-term outcomes has not yet been shown, an association has been shown between acetaminophen use during pregnancy and risk for developmental outcomes in children. Perinatal health care providers should provide mothers with adequate information about the fact that acetaminophen is readily transferred to breast milk.

## Data Availability

The data presented in this study are available on request from the corresponding author. The data are not publicly available due to privacy.

## Abbreviations

AUC: Area under the curve
ADHD: Attention-deficit/hyperactivity disorder
HKD: Hyperactivity disorder
LC-MS/MS: Liquid chromatography-electrospray ionization tandem mass spectrometry
M/P ratio: Milk-to-plasma drug concentration ratio
RID: Relative infant dose
